# UV polymerization fabrication method for polymer composite based optical fiber sensors

**DOI:** 10.1038/s41598-023-33991-6

**Published:** 2023-07-04

**Authors:** Israr Ahmed, Murad Ali, Mohamed Elsherif, Haider Butt

**Affiliations:** 1 Department of Mechanical Engineering, Khalifa University of Science and Technology, Abu Dhabi, 127788 UAE; 2 Division of Engineering, New York University, Abu Dhabi, UAE

**Keywords:** Materials for devices, Materials for optics, Soft materials

## Abstract

Optical fiber (OF) sensors are critical optical devices with excellent sensing capabilities and the capacity to operate in remote and hostile environments. However, integrating functional materials and micro/nanostructures into the optical fiber systems for specific sensing applications has limitations of compatibility, readiness, poor control, robustness, and cost-effectiveness. Herein, we have demonstrated the fabrication and integration of stimuli-responsive optical fiber probe sensors using a novel, low-cost, and facile 3D printing process. Thermal stimulus–response of thermochromic pigment micro-powders was integrated with optical fibers by incorporating them into ultraviolet-sensitive transparent polymer resins and then printed via a single droplet 3D printing process. Hence, the thermally active polymer composite fibers were grown (additively manufactured) on top of the commercial optical fiber tips. Then, the thermal response was studied within the temperature range of (25–35 °C) and (25–31 °C) for unicolor and dual color pigment powders-based fiber-tip sensors, respectively. The unicolor (with color to colorless transition) and dual color (with color to color transition) powders-based sensors exhibited substantial variations in transmission and reflection spectra by reversibly increasing and decreasing temperatures. The sensitivities were calculated from the transmission spectra where average change in transmission spectra was recorded as 3.5% with every 1 °C for blue, 3% for red and 1% for orange-yellow thermochromic powders based optical fiber tip sensors. Our fabricated sensors are cost-effective, reusable, and flexible in terms of materials and process parameters. Thus, the fabrication process can potentially develop transparent and tunable thermochromic sensors for remote sensing with a much simpler manufacturing process compared to conventional and other 3D printing processes for optical fiber sensors. Moreover, this process can integrate micro/nanostructures as patterns on the optical fiber tips to increase sensitivity. The developed sensors may be employed as remote temperature sensors in biomedical and healthcare applications.

## Introduction

Optical fibers are traditionally flexible, transparent, and cylindrical waveguides that deliver or guide light for long distances with minimum optical losses^1–3^. Their fine dimensions allow them to be integrated with medical devices, such as catheters and needles, for in vivo sensing, point-of-care testing, examining small samples, and accessing confined spaces^4^. Therefore, optical fiber sensors have been essential for sensing applications in various disciplines, especially in biomedical applications due to their compactness, flexibility, chemical inertness, biocompatibility, and ability to be machined and functionalized. Furthermore, these sensors can independently monitor one or more parameters such as temperature, humidity, and molecular species concentration even in harsh environments^1,5^.

Thermal sensing is vital in various industries such as automobiles, robotics, petrochemical, power generation, agriculture, medical diagnostics, textile and clothing, and smart sensing^6–10^. Therefore, application-specific temperature sensors are fully developed and applied in numerous applications to maintain suitable working conditions and to avoid any breakdowns or failures. For instance, continuous temperature monitoring assists in maintaining the well-being of aquatic organisms, mainly various types of fish and insects^11^. Temperature sensors control the rate of reaction and solubility of various materials in chemical industries such as in the conversion to green fuels^12^. Moreover, such sensors are also suitable for thermal sensing in the textile and clothing industry which detect temperature changes from visual color transformation^10^. Most types of temperature sensors used are electrical, but these are unable to perform in harsh environments and may lead to erroneous results due to their susceptibility to electrical and magnetic interferences. The alternative and more suitable solution are polymer-based optical fiber sensors which have strong immunity to electromagnetic interferences and can be employed in remote sensing^6,13^. The reported optical fiber temperature sensors based on hybrid glass fibers^14^, interferometers^15,16^, and fiber Bragg gratings^17,18^ have already shown high sensitivity but are susceptible to small mechanical and environmental disturbances. However, polymer-based optical fiber temperature sensors have adequate strength, flexibility, negative thermos-optics coefficient, multiplexing capabilities, and minimum environmental interactions on the performance at ambient conditions making them an alternative solution^19,20^. Thus, their low sensitivity to external disturbances makes them more accurate and reusable^21,22^. Additionally, polymer materials have exceptional compatibility with organic materials, offering them ubiquitous role in clinical applications. In the past, multimode polymer-based optical fiber sensors have been effectively used to take advantage of these characteristics^20^.

The common fabrication processes for optical glass fibers from their preforms are Vapor Phase Oxidation (VPO), Axial Deposition (AD), Modified Chemical Vapor Deposition (MCVD), and Plasma-activated Chemical Vapor Deposition (PACVD)^23^. Standard glass fibers are typically produced then by “preform heat and draw” using fiber drawing towers. Similarly, polymer-based optical fibers can be fabricated using their preforms via drawing at above their glass transition temperatures making the process similar to single-mode fiber fabrication process^24^. Recently, the rise of additive manufacturing or 3D printing shifted this paradigm to more complex and customized fabrication of optical devices. These optical devices can work independently or be part of complex optical systems^25–28^. Therefore, latest 3D printing developments are playing a revolutionary role in sensing and analytical optical systems^29–31^. A large variety of optical materials including metallic, ceramic, polymeric, and their composites are available and can be customized to targeted specific applications^32–34^. Currently, the most promising available 3D printing techniques for polymer-based optical fibers are Fused Filament Fabrication (FFF)^35^, Stereolithography (SLA), Masked Stereolithography (MSLA)^6^, and Digital Light Processing (DLP)^36^. The freeform of soft glassy and polymer optical fiber has already been printed via the FFF technique that was pulled out afterward to make optical fibers^37,38^. However, FFT is more suitable for thermoplastic materials while SLA, MSLA, and DLP are used to print 3D structures using photocurable resin materials^6,39^. Recently, 3D printed single droplet freestanding structures without support have been reported. Although they have printed curved structures like optical fibers without a specific application and integration with optical fibers but have better dimension control due to the multi-axis pneumatic printing system^40^. In short, these printing techniques utilize a wide range of customized printable materials, allowing them extra functionalities by exploring specific material compositions.

The 3D printing techniques mentioned earlier are promising for fabricating optical fibers based on technological advancements in the printing industry. Despite that, these printing techniques have some advantages or disadvantages when employed for specific applications. For instance, SLA cures 3D structure point-by-point consuming more time compared to MSLA and DLP printing techniques where an entire resin layer is cured at once. Similarly, post-processing steps, such as lacquering is essential for SLA, MSLA, and DLP printed parts while FFF printed parts might need extra polishing to achieve the finished product requirements. Recently, pixelate effect has been reported to influence the optical performance of MSLA printed parts. However, MSLA is comparatively faster than SLA and has high resolution. Similarly, FFF offers lower z resolution while DLP provides less z x–y resolution than MSLA^6,29,41^.

All these printing techniques require a substantial resin material volume, even for small parts to initiate the printing process. None of these techniques can be employed on a single droplet for printing the desired optical fiber or for printing sophisticated functionalized optical fiber tips. Also, these processes have a limitation of integrating the printed fibers with the commercial optical fiber systems. Moreover, layer-by-layer printing by these techniques results in many interfaces, even for small structures, leading to reduced optical performance. Therefore, a printing method that can adequately address these issues is essential. In this work, we demonstrate a novel single droplet 3D printing process for optical fibers and fiber tip fabrication, with inherent physical adhesion leading to better integration with commercial optical fiber systems. Optical fibers and functionalized fiber tips with reasonable length can be manufactured using this process. Fiber-tip functionalization can be achieved by using functional resin materials including thermochromic pigment, colored pigment, etc., as well as nanoimprinting micro/nano surface structures such as Aztec nanostructure^42^. Furthermore, the fabrication process is quite simple, straightforward, and cost-effective.

Herein, transparent optical fiber and thermally sensitive optical fiber tips were fabricated via single droplet 3D printing technique for thermal sensing applications. Reversible thermochromic micro-powders were used to add thermal sensing functionality into photocurable resin comprised of polyhydroxyethyl methacrylate (p-HEMA) and polyethylene glycol diacrylate (PEGDA) based polymer fibers. Thermochromic pigment powders are non-toxic and thermally active exhibiting visual color transformation reversibly upon heating and cooling cycles^6,43,44^. The thermochromic pigment powders are of two types, unicolor and dual color. Unicolor pigment powders go through color to colorless transition (blue and red to transparent) reversibly upon heating and cooling, while dual color powders undergo color to color transition (orange to yellow) reversibly upon heating and cooling. The unicolor and dual color powders have temperature ranges of 25–35 °C and 25–31 °C, respectively. The 3D printed optical fibers and fiber tips were investigated for their physical, microstructural, and optical properties. The thermal sensing functionality was quantitatively assessed in their respective temperature range to show their ability as temperature sensors.

## Results and discussion

**Figure 1:**
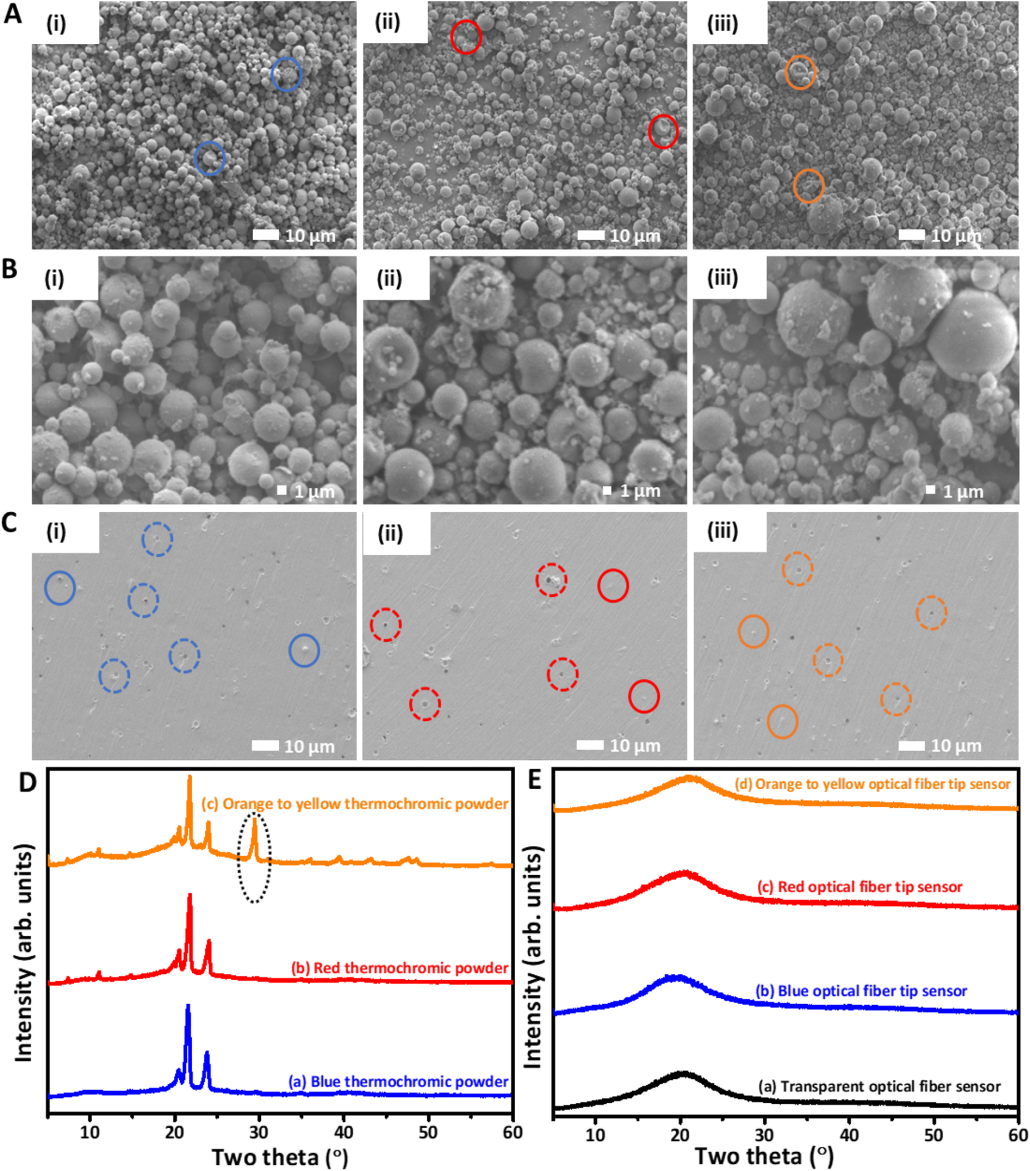
Morphological, microstructural, and physical characterizations of thermochromic powders HEMA resin, and 3D printed optical fibers. (**A**) SEM micrographs of different thermochromic pigment powders are shown as (i) blue, (ii) red, and (iii) orange to yellow. (**B**) The magnified SEM micrographs of respective thermochromic powders in figure (**A**). (**C**) SEM micrographs of cross-sectional views of mechanically fractured 3D printed thermochromic sensors are represented as (i), (ii), and (iii) in the figure. (**D**) XRD patterns of thermochromic powders are presented as (a) blue, (b) red, and (c) orange to yellow. (**E**) XRD patterns of 3D printed fiber sensors are presented as (a) transparent, (b) blue, (c) red, and (d) orange to yellow in the figure.

The morphological investigation of micron-sized thermochromic powders and 3D printed thermochromic optical fiber tip sensors was performed using SEM (Fig. 1A-C(i–iii)). The SEM micrographs reveal the spherical morphology of the powders with diameters range of ~ 1–5 µm (Fig. 1A(i-iii). The SEM micrographs also revealed some shell deformation (highlighted by solid circles with their respective colors) of spherical powders which appeared to have a well-defined core–shell structure^6,43^. In the relatively magnified micrographs, three thermochromic powders exhibited the same morphological characteristics (Fig. 1B(i–iii)). As prior to 3D printing, micro-powders were added to the HEMA-based resin matrix; therefore, it’s paramount to examine how they were distributed throughout the 3D printed samples. Thus, pigmented samples were first mechanically fractured, and then their cross-sections were studied using SEM. The micrographs revealed homogeneous distribution of micro-sized pigment powders without visible agglomeration. As a result of mechanical fracture, some particles were pulled out from the matrix shown by dotted circles with their respective colors, while others were still embedded in the HEMA resin matrix (Fig. 1C(i–iii)). The smooth surface of the cross-sectioned samples also demonstrates the integrity of the printing process by depicting no visible pores or cavities affecting the fabrication process. Moreover, pigment powder particles retain their shapes during the printing/polymerization process, and therefore, similar behavior was observed in SEM micrographs.

X-ray diffraction analysis was carried out for the thermochromic powders and their respective 3D printed fiber samples to determine their crystallinity and any other new phase formation during photopolymerization in the printing process Fig. 1D,E. Three sharp peaks of unicolor and dual color thermochromic powders were present at 2θ of ~ 20.5°, ~ 21.5° and ~ 23.7° in the XRD pattern confirming their crystalline nature. However, dual color powder (orange to yellow) showed an extra peak at 29.43° which could be attributed to its different synthesis method or structural identity in Fig. 1D^45^. The XRD pattern of HEMA-based resin without thermochromic powders consisted of a broad peak indicating the amorphous nature of the transparent printed sensor. However, 3D printed thermochromic samples exhibited the same characteristic broad peak, and no additional peaks of added colored powders were observed. The missing peaks might be due to the XRD machine’s detection limit of 2 vol.%^6,46,47^. Below this limit, peaks of material mix with background noise and become indistinguishable. Hence, colored pigment powders were added in 1 vol.%, which justified the disappearance of their peaks from acquired XRD patterns. Based on XRD findings, no additional phases were formed during the single droplet 3D printing process Fig. 1E.

**Figure 2:**
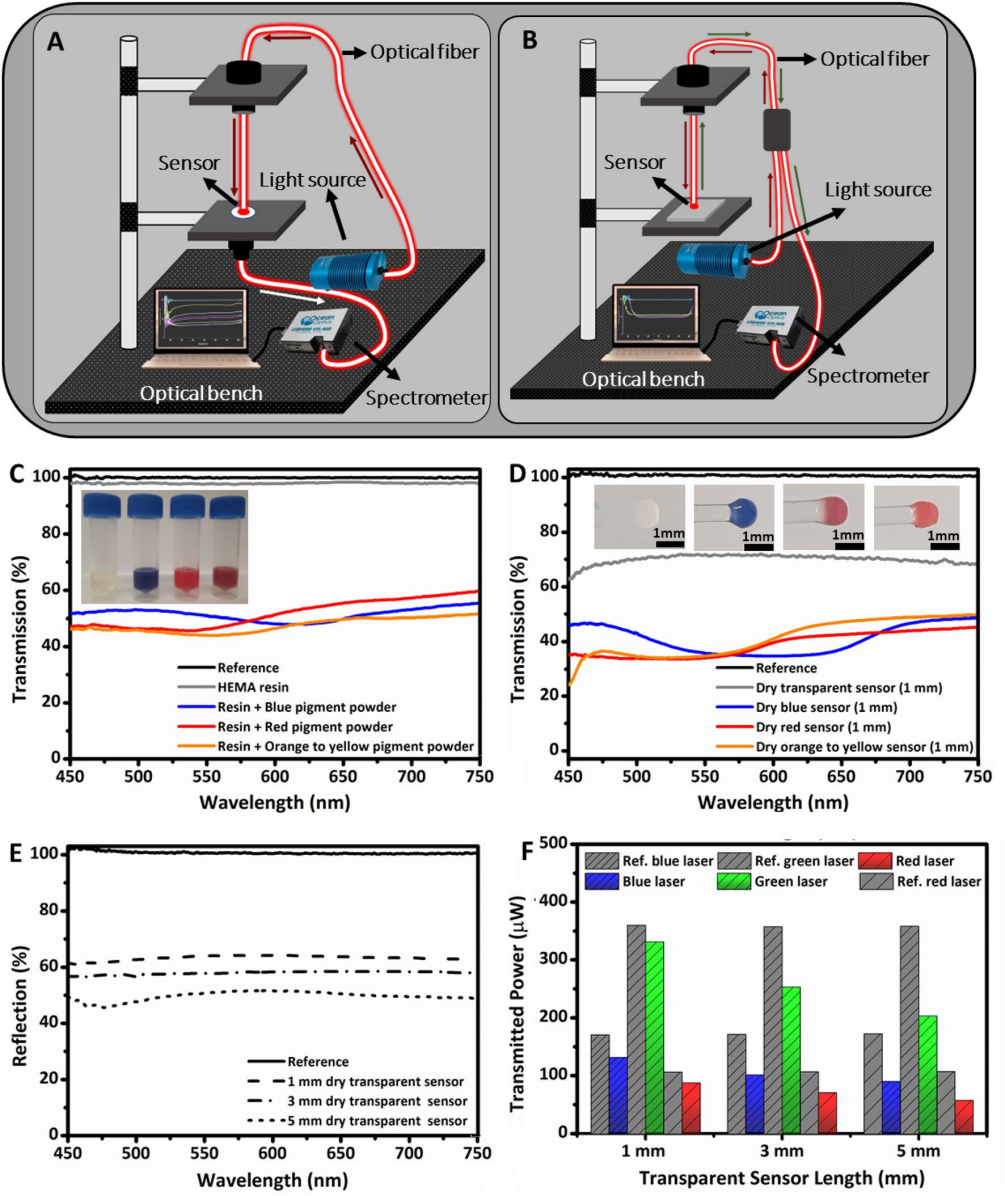
Optical characterization of HEMA resin, resin-powder mixtures, and 3D printed optical fiber sensors using customized experimental setups. (**A**) Schematic diagram to measure the optical transmission of the printed sensors and (**B**) schematic illustration of setup used to measure the optical reflection of printed transparent fiber sensors. (**C**) Transmission spectra of liquid resin and resin-powder mixtures using an optical microscope, (**D**) transmission spectra of printed transparent and thermochromic fiber tip sensors, and (**E**) reflection spectra of printed transparent sensors. (**F**) Transmitted optical power measurements of printed transparent sensors of varying lengths using blue (405 nm), green (532 nm), and red (650 nm) lasers.

Transmission and reflection are key characterizations for investigating the optical properties of HEMA resin, resin-powder mixtures, and printed optical fiber sensors. The schematic diagrams of experimental setups are presented in Fig. 2A,B while the results are analyzed in Fig. 2C–E. A glass slide was used as a reference for resin-powder mixtures, whereas commercial optical fiber was used as a reference for the printed sensors. Moreover, digital images of insets in Fig. 2C,D present colored mixture solutions and their respective 3D printed sensors. The transmission spectra of resin-powder mixtures and 3D printed sensors remain unchanged which indicates that no chemical interaction occurred between pigment powders and HEMA resin during physical mixing or 3D printing based on photopolymerization (Fig. 2C,D). However, transmission spectra of all resin-powder mixtures contain specific dips corresponding to their blue, red, and orange colors. Moreover, resin-powder mixtures have similar transmission intensities range (% transmission) between 450 to 750 nm wavelengths. Comparatively, transmission intensities of materials including transparent resin and resin-powder mixtures were reduced after 3D printing. The highest reduction in average transmission (~ 22% ↓) was observed for transparent sensors, followed by colored tip sensors (~ 5–10% ↓) (Fig. 2C,D). Since the transmission of 3D printed sensors primarily depends on their thicknesses and coloration^48^. This also might be attributed to light interaction with chemical structures based on their bonding during the phase transformation from liquid to solid state upon photopolymerization. Moreover, typical 3D printing processes based on photopolymerization require post-curing, leading to a visual effect called “yellowing” in final samples^49,50^. However, no such effect in our printed transparent fibers was observed, possibly due to no requirement of post-processing in our fabrication process. Reflection spectra were also recorded for 3D printed transparent optical fibers with varying lengths of 1, 3, and 5 mm. The highest average transmission (~ 63%) was observed for optical fiber with the shortest length of 1 mm. The average transmission was gradually reduced to ~ 58% and ~ 50% with the increase in length of optical fibers to 3 and 5 mm, respectively (Fig. 2E). The reduction in transmission intensities might be attributed to interfaces formed during the fabrication process due to manual control.

A customized experimental setup was used to measure the transmitted power intensities through the transparent optical fibers of 1, 3, and 5 mm lengths. The schematic illustration of experimental setup is shown in Fig. S2 (Supporting Information)**,** and the results are provided in (Fig. 2F). Transparent optical fiber sensors were placed 2 mm below the power meter to achieve consistent and comparable results. Each transparent sensor transmitted lower power than its respective reference fiber indicating less power intensity transmission compared to silica material of commercial optical fiber. However, transmitted power showed a decreasing trend with the increased sensor length for all the printed transparent sensors. The power reduction was maximum for green laser (~ 128 µW), followed by blue (~ 41 µW) and red (~ 30 µW) lasers for increasing sensor length from 1 to 5 mm. Among the lasers, maximum power was transmitted for the green laser, and this might be due the intrinsic absorption properties of the resins used (Fig. 2F). Thus, green laser is more suitable for experimenting with transparent printed sensors.

**Figure 3:**
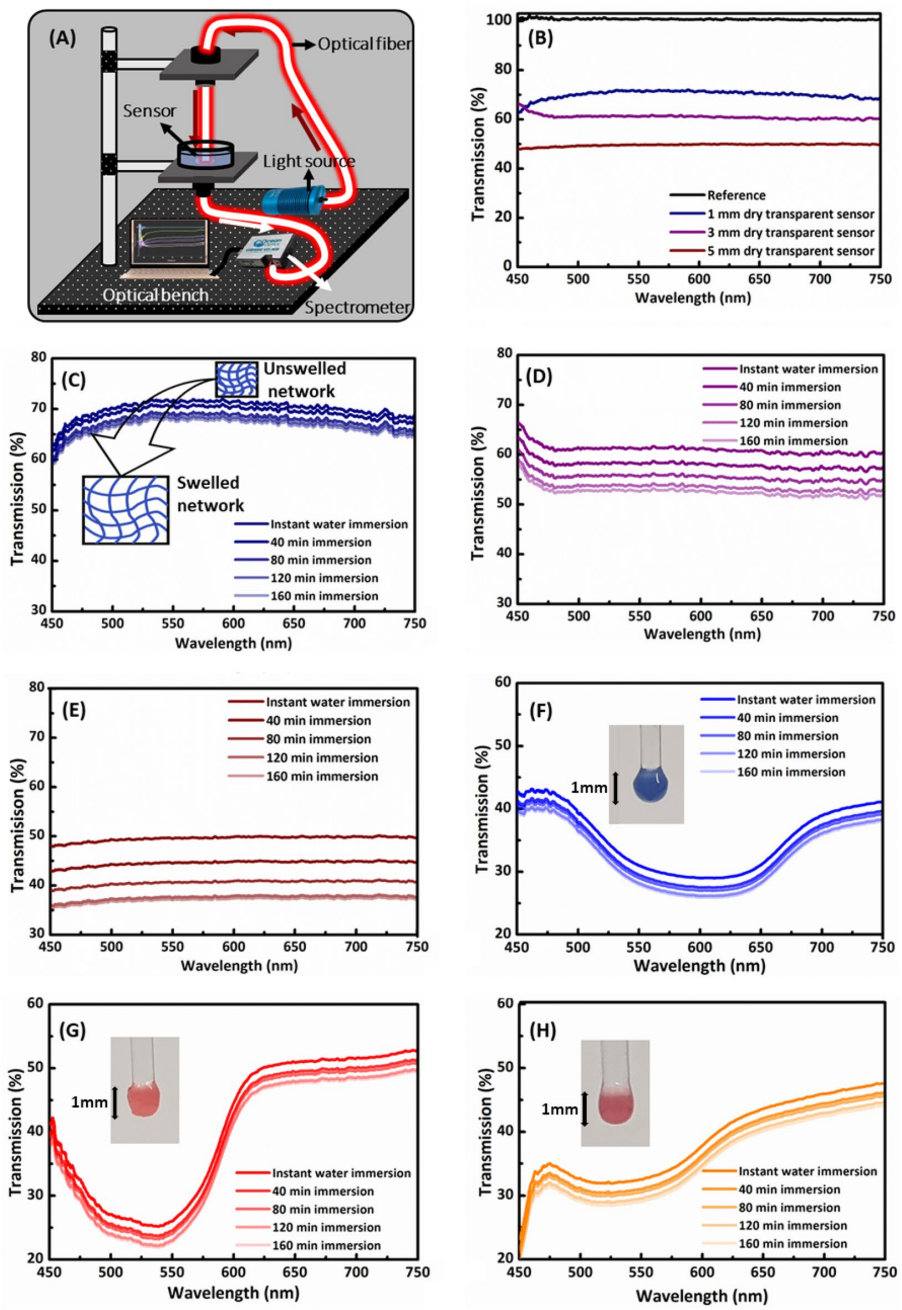
Dynamic transmission analysis of 3D printed transparent and thermochromic fiber tip sensors. (**A**) A schematic illustration of the customized optical setup for transmission measurements. (**B**) Transmission spectra of 3D printed transparent optical fiber sensors with different lengths of 1 mm, 3 mm, and 5 mm. The transmission spectra investigate the swelling behavior of transparent fiber sensors of varying lengths; (**C**) 1 mm, (**D**) 3 mm, and (**E**) 5 mm. Transmission spectra of 1 mm optical fiber tip sensors with different thermochromic powders; (**F**) blue, (**G**) red, and (**H**) orange to yellow.

Dynamic transmission analysis investigated the swelling behavior of the hydrogel-based manufactured optical sensors. The schematic diagram of the customized setup for a combined transmission and swelling behavior is presented in Fig. 3A. First, transmission spectra of 3D printed transparent optical fiber sensors having 1, 3 and 5 mm lengths were obtained to analyze them in dry conditions, i.e. without DI water (Fig. 3B). As expected, the highest average transmission of ~ 70% was observed for 1 mm length transparent sensor, followed by 3 mm (~ 61%) and 5 mm (~ 50%) sensors, respectively. Overall, a decreasing trend in optical transmission was observed with the increase in sensor length (Fig. 3B). Second, these printed transparent sensors of 1, 3, and 5 mm were individually characterized for their swelling characteristics in DI water using transmission analysis (Fig. 3C–E). Several studies have reported the swelling behavior of p-HEMA in aqueous solutions. In dry conditions, p-HEMA is transparent, but morphological and microstructural changes occur after submerging in water. Above the critical water contents, pores formation in hydrogels occurs resulting in a loss in transparency that can be attributed to phase separation between p-HEMA and water. These pores are small in dry state but grow larger when hydrogels imbibe water and act as visible light scattering centers. In addition to pores, the scattering from pores is higher for high refractive contrast; thus, loss in transparency may be partly due to increased refractive index contrast of the p-HMEA during soaking in water^51^. Similar behavior of loss in the transparency of the printed transparent fiber sensors of different lengths has been observed **(**Fig. 3C–E**)**. For each sensor, transmission spectra at specific time intervals of instant immersion (2), 40, 80, 120 and 160 min were recorded with respect to wavelength. The instant immersion time interval represents recorded transmission spectra of sensors in about 2 min after submerging in DI water. A gradual increase in immersion time with 40 min step increment resulted in gradual decrease in the average optical transmission of ~ 5%, ~ 9%, and ~ 12% for 1, 3, and 5 mm sensors, respectively. Interestingly, 1 mm transparent sensor yields less variations (5%) in transmission spectra than 3 and 5 mm sensors over the entire immersion period. Therefore, variations in transmission spectra followed an increasing trend with an increase in sensor length, and each sensor was saturated after 160 min of immersion. Similarly, the swelling behavior of thermochromic optical fiber tips was investigated (Fig. 3F–H). The pigmented tip sensors repeatedly exhibited their characteristic dips in transmission spectra based on their colors (blue, red, and orange to yellow) even during soaking in DI water. The transmission spectra of pigmented tip sensors shifted to lower transmittance by less than 5% (average value) over the immersion period due to swelling and reached saturation level at 160 min. The variations in transmission spectra of pigmented sensors was less compared to the transparent samples, and this might be attributed to small thermochromic powder particles in the swollen hydrogel. Moreover, due to the same size of the tip sensors, variations in their transmission spectra were also similar (Fig. 3F–H).

The thermal sensing functionality of the fabricated tip sensors was assessed based on their color transformation due to temperature changes in an aqueous environment. Deionized water was used to ensure homogenous heat distribution around the sensors during the heating process. The increase or decrease in DI water’s temperature (reversible process) incurred variations in the colors of pigmented sensors. The unicolor powders were visible as blue and red at room temperature of 25 °C and then turned transparent at high temperature of 35 °C. However, dual color powder appeared orange at room temperature of 25 °C and then yellow at high temperature of 31 °C. The response time in the air is of few seconds but was slowed down and controlled in DI water to visualize each color variations at a specific temperature properly. The reusability of developed sensors is critical for their applications in targeted areas such as remote sensing. Therefore, multiple heating and cooling cycles were applied to fabricated optical fiber tip sensors to ensure the repeatability and durability of their thermosensitive feature. Moreover, developed sensors were repeatedly tested in DI water, and no sign of pigment leaching out was observed, confirming the integrity of the 3D printing process. The experimental setups of transmission and reflection measurements utilized to conduct these experiments for blue, red, and orange to yellow thermally activated powders are presented in Figs. S3 and S4 (Supporting Information).

**Figure 4:**
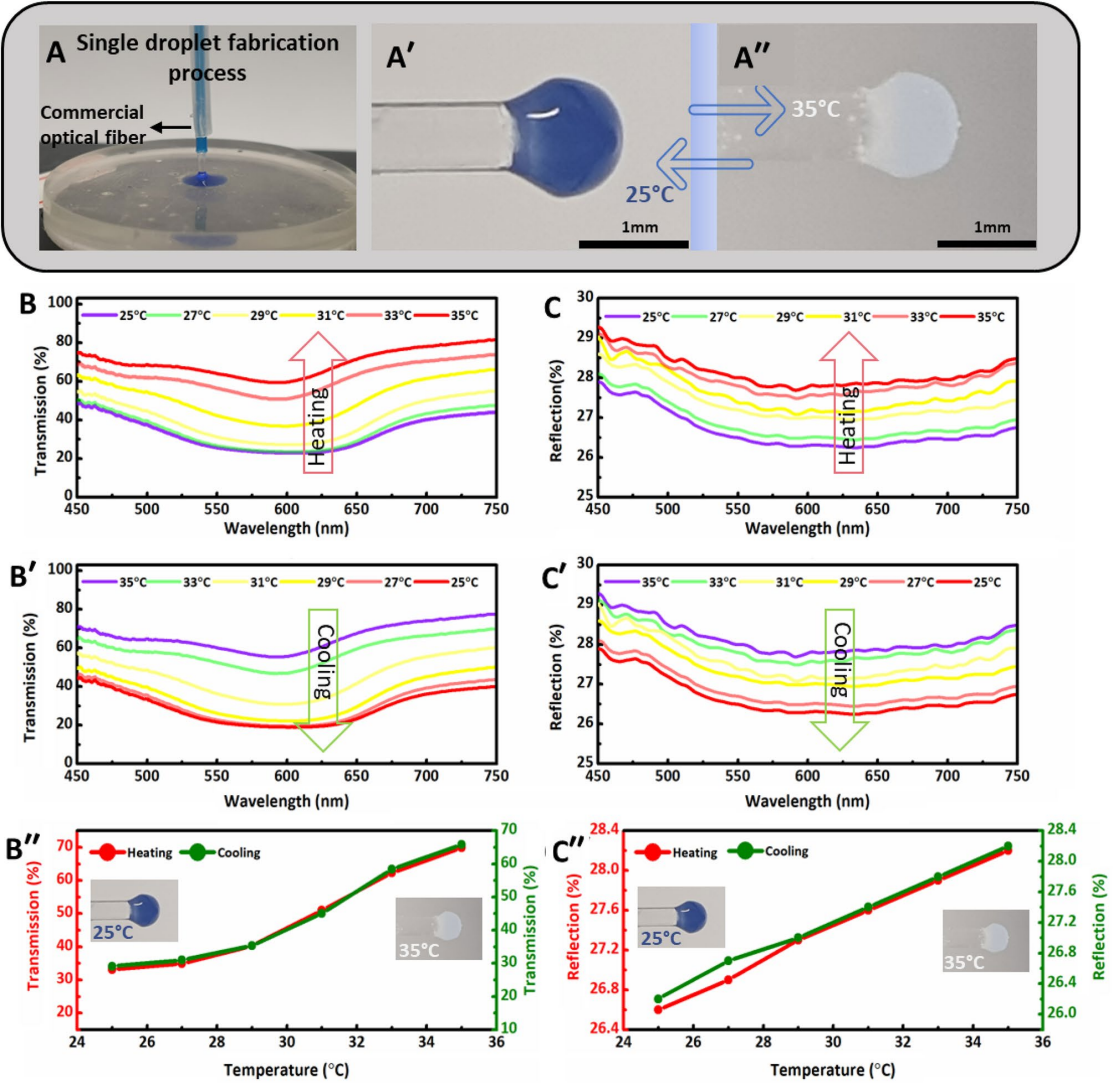
Fabrication, light transmission, and reflection spectra of developed blue thermochromic optical fiber tip sensor. (**A**) Fabrication of blue thermochromic sensor from the respective resin-powder mixture. Color transformation of the developed blue sensor at different temperatures: (**A′**) 25 °C and (**A′′**) 35 °C. The transmission and reflection spectra upon heating and cooling were indirectly recorded with respect to wavelength in the 25–35 °C temperature range with 2 °C steps. Transmission spectra for blue sensor upon (**B**) heating and (**B′**) cooling. (**B′′**). Average transmission intensities values during heating and cooling cycles from each curve at different temperatures in the wavelength range of 450–750 nm. Reflection spectra of blue sensor upon (**C**) heating and (**C′**) cooling. (**C′′**) Average reflection intensities values from each curve during heating and cooling at various temperatures in the wavelength range of 450–750 nm.

The thermal response of 3D printed blue thermochromic optical fiber tip sensor was assessed using dynamic transmission and reflection analyses (Fig. 4). Figure 4A shows the developed (blue ↔ transparent) sensor, where the curing light travels through the optical fiber into the blue resin-powder mixture drop and cured it on the tip of commercial optical fiber. The fabricated blue tip sensor was tested, and the response was captured in digital images at 25 °C and 35 °C. The tip sensor appeared blue at room temperature of 25 °C in air and aqueous environments (Fig. 4A′). Since the tip sensor was tested in an aqueous environment, the accumulation of pores in hydrogels due to water absorption acted as scattering centers that led to translucent appearance as shown in (Fig. 4A′′). However, the tip could be dried to avoid the translucent effect by heating at 60 °C for 20 min. The transmission spectra of the blue tip sensor were recorded when subjected to heating and cooling cycles in the temperature range of 25–35 °C with 2 °C steps (Fig. 4B,B′). The transmission spectra during heating and cooling were monitored and measured using an attached portable thermometer. The characteristic dip of blue color in the transmission spectrum gradually vanished upon increasing the temperature to 35 °C in an aqueous environment and became like transparent fiber. Thus, the blue sensor transmitted more light in the 450–750 nm range with an increase in temperature due to the blue color transformation to transparent. However, the exact flatness of dip was hindered due to translucent effect, and complete flatness of dip could be achieved by maintaining water contents below the critical contents limit in hydrogels above which light scattering pores appear. A strong positive correlation between transmission intensity and temperature was observed. As a result, the average transmission intensity of the sensor increased with the increase in temperature due to less optical absorption of blue micro-particles during the heating cycle (Fig. 4B′′). Similarly, transmission intensity reduced with decreasing temperature during the cooling cycle, and the digital images of the sensors at low and elevated temperatures are also shown in the insets of Fig. 4B′′.

Again, thermal sensing analysis was performed for the blue tip sensor using another customized experimental setup to measure the reflection mode spectra (Fig. S4, Supporting Information**)**. The reflection spectra of the blue sensor were recorded against wavelength in the 25–35 °C temperature range with 2 °C steps for both heating and cooling cycles (Fig. 4C,C′). A similar trend of flatness in reflection curves was observed with an increase in temperature, and maximum flatness was achieved at 35 °C (Fig. 4C). Evidently, variations in reflection spectra (~ 2%) with temperature change were less compared to transmission spectra (~ 36%) during cooling and heating cycles. However, the blue sensor qualifies for testing in different environments using transmission and reflection modes-based sensing mechanisms. The reflection curve intensities were restored to the original blue color during the cooling cycle to room temperature (Fig. 4C′). The average values of each reflection curve to respective temperature during the heating and cooling cycle are presented in (Fig. 4C′′). The less absorption exhibited by the blue thermochromic powder at high temperatures, enhanced the reflection intensity. At high temperature the powders displayed a whitish tint, which signifies optical scattering in all directions, and hence improved backscattering and reflection intensity.

**Figure 5:**
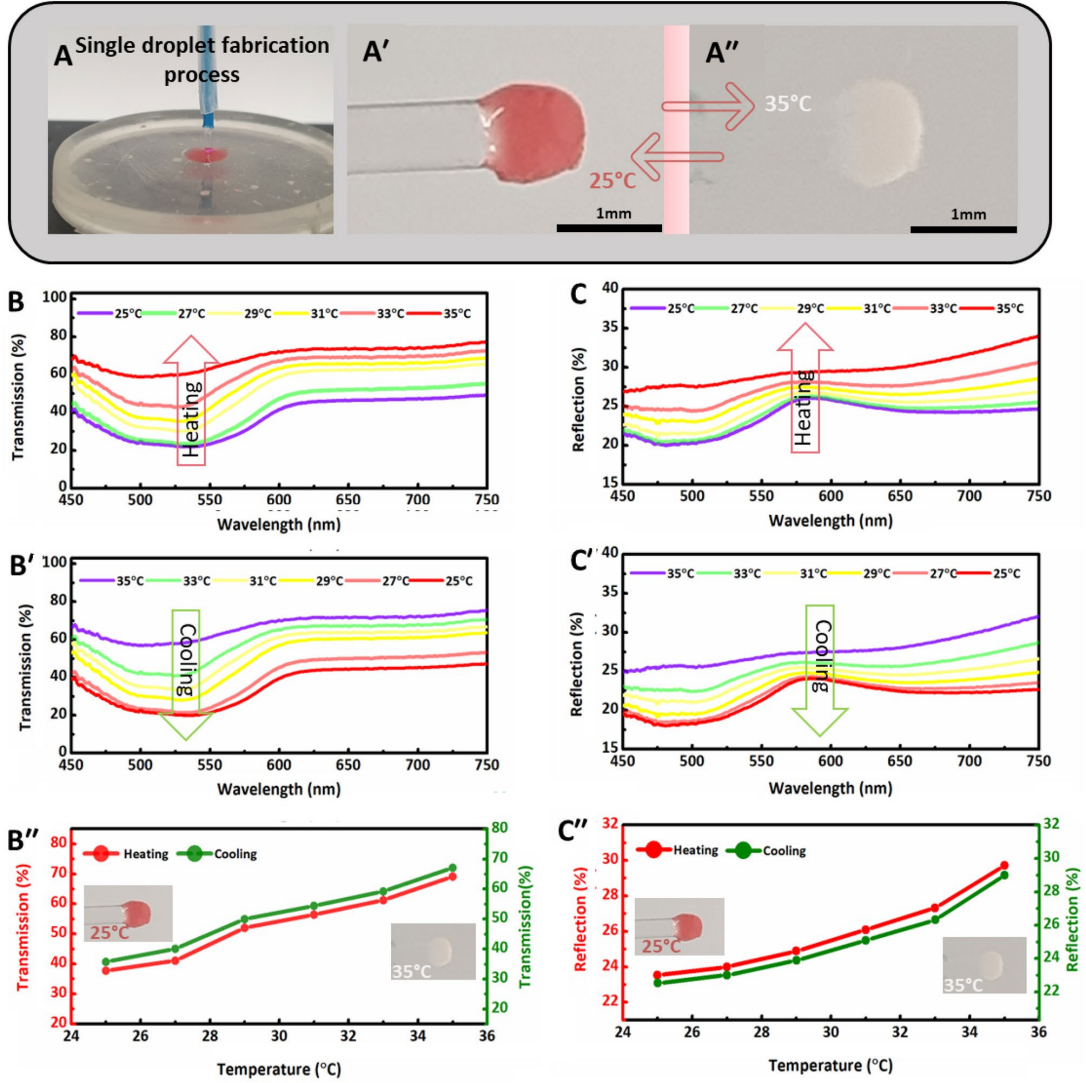
(**A**) Fabrication process of red thermochromic optical fiber tip sensor from the respective resin-powder mixture and color transformation at different temperatures: (**A′**) 25 °C and (**A′′**) 35 °C. The transmission and reflection spectra were indirectly recorded with respect to wavelength during heating and cooling cycles in the 25–35 °C temperature range with 2 °C steps. Transmission spectra for red tip sensor upon (**B**) heating and (**B′**) cooling. (**B′′**). Average transmission intensities values during heating and cooling cycles from each curve at different temperatures in the wavelength range of 450–750 nm. Reflection spectra of red sensor upon (**C**) heating and (**C′**) cooling. (**C′′**) Average reflection intensities values from each curve during heating and cooling at various temperatures in the wavelength range of 450–750 nm.

The thermal sensing functionality of the red thermochromic optical fiber tip sensor was studied to ensure the compatibility of the 3D printing process with different photocurable materials. The fabrication process and thermal response of the developed sensor (red ↔ transparent) are shown in digital images (Fig. 5A–A′′). The transmission spectra during heating and cooling cycles were recorded at different temperatures between 25 and 35 °C. The 2 °C incremental increase in temperature to 35 °C (heating cycle) resulted in similar curve flatness, also observed for the blue sensor (Fig. 5B). A gradual decrease in temperature to 25 °C (cooling cycle) ensured restoration to the original red color of the sensor as presented in Fig. 5B′. The average values of transmission intensities from each curve during cooling and heating cycles with respective temperatures are plotted in Fig. 5B′′. With the increase in temperature, transmission intensity increased due to low light absorption of red micro-particle, indicating a positive correlation between transmission intensity and temperature. The reflection spectra analysis was also performed for the red tip sensor by recording reflection spectra during heating and cooling cycles in the temperature range of 25–35 °C, with 2 °C steps. A similar trend in reflection curve flatness with temperature variation was observed compared to the blue sensor (Fig. 5C,C′). Moreover, average values of intensities from reflection curves increased with increase in temperature to 35 °C due to sensor transformation from red to transparent state (Fig. 5C′′). Interestingly, it is observed that the red fiber tip sensor has a reflection peak near 580 nm, signifying that it preferentially reflects red colors at lower temperatures. Also, the transmission graphs show that the sensor has a dip centered around 525 nm. Signifying that at lower temperatures the powder absorbs green–blue color more than the red colors. At high temperatures when the powder is white, all colors are more or less uniformly absorbed and reflected.

**Figure 6:**
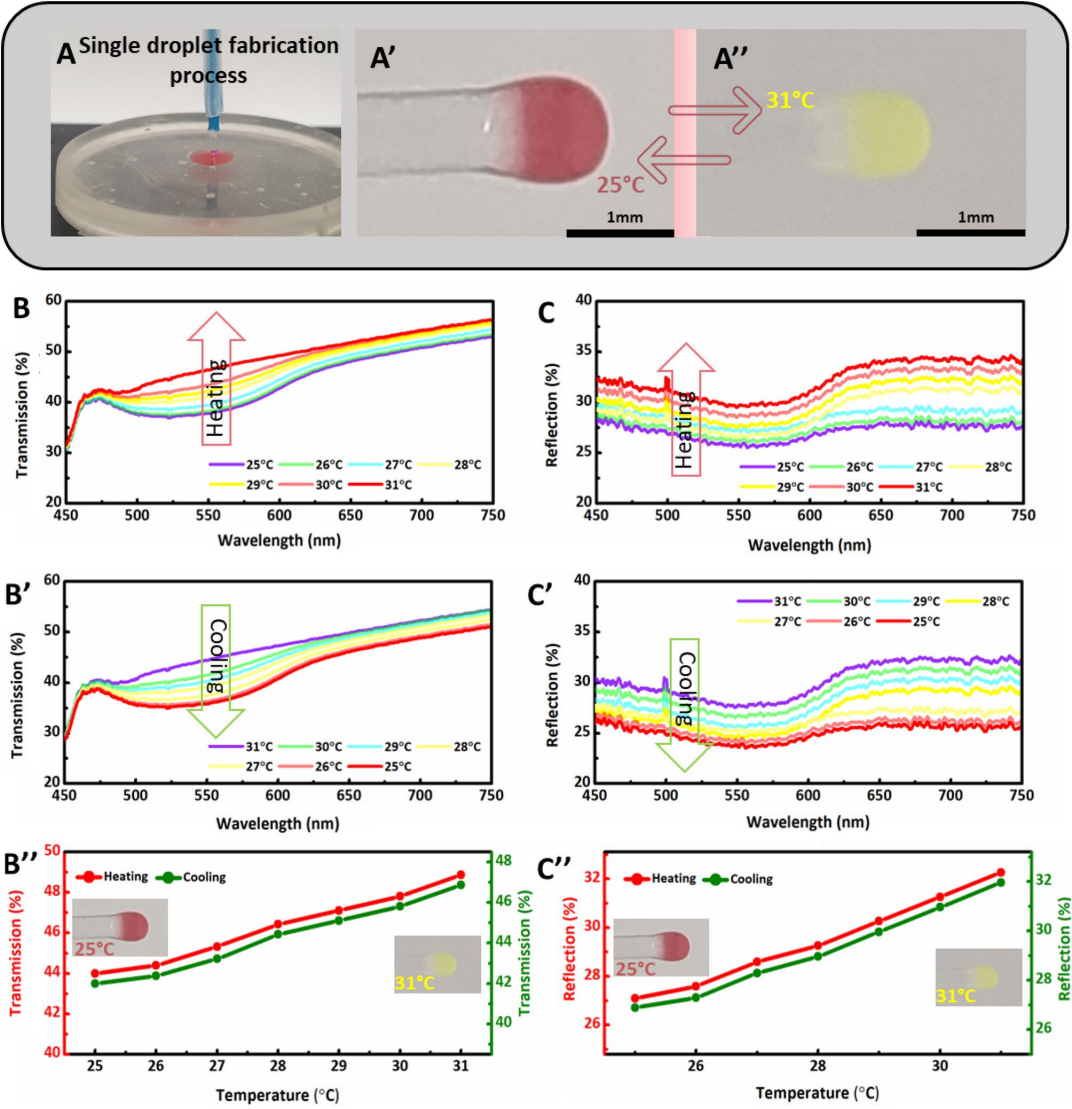
(**A**) Fabrication process of orange-yellow thermochromic optical fiber tip sensor from the respective resin-powder mixture and color transformation at different temperatures: (**A′**) 25 °C and (**A′′**) 31 °C. The transmission and reflection spectra were indirectly recorded with respect to wavelength during heating and cooling cycles in the 25–31 °C temperature range with 1 °C steps. Transmission spectra for orange-yellow tip sensor upon (**B**) heating and (**B′**) cooling. (**B′′**). Average transmission intensities values during heating and cooling cycles from each curve at different temperatures in the wavelength range of 450–750 nm. Reflection spectra of the sensor upon (**C**) heating and (**C′**) cooling. (**C′′**) Average reflection intensities values from each curve during heating and cooling at various temperatures in the wavelength range of 450–750 nm.

Wavelength-selective temperature sensors have various applications in energy harvesting and remote and perceptive sensors in various devices. An attempt has been made to achieve a 3D printed wavelength-specific thermal optical fiber tip sensor using dual color thermochromic pigment powder in the resin matrix. The dual color pigment goes through color to color (orange ↔ yellow) transition in the temperature range of 25–31 °C. The single droplet based 3D printing process and the resulting fiber tip sensor’s thermal transition, between orange and yellow colors, are shown in (Fig. 6A–A′′). The transmission spectra of orange to yellow sensor were recorded sensor are also presented in insets (Fig. 6B,B'). The reflection spectra of the dual color tip sensor step size of 1 °C. At room temperature (25 °C) tip sensor was orange, and the color transition to yellow occurred at 31 °C. The characteristic dips of both colors change with temperature variations based on specific concentrations utilized in this work (Fig. 6B,B′). The average transmission intensity values obtained from each curve were plotted against their respective temperatures which showed significant change in transmission intensities with the color transformation as shown in (Fig. 6B′′). The reflection spectra of the color transition from orange to yellow of the developed tip were also recorded with respect to the wavelength in the temperature range of 25–31 °C during heating and cooling cycles (Fig. 6C,C′). Finally, the average values of reflection intensities from each curve were plotted against their respective temperatures (25–31 °C), and variations in intensities are evident (Fig. 6C′′).

The recent advances in smart materials have inspired researchers to pave the way toward new possibilities. Thermochromic materials and their polymeric composites are in high demand due to their excellent optical, storage, and color-changing properties in response to the external stimulus such as temperature. According to the experimental findings, thermochromic optical fibers manufactured using novel 3D printing can measure temperature changes in remote environments of liquids with excellent precision over a limited temperature range of (25–35 °C) for unicolor and (25–31 °C) for dual color thermochromic powders.

The two crucial parameters that must be addressed and discussed for every developed sensor are resolution and sensitivity. Resolution refers to the smallest possible difference in measurements that the sensor can detect, while sensitivity is the minimum amount of change in the measured parameter required to produce a reading change equivalent to the sensor's resolution. By carefully considering both of these parameters during the testing process, a detectable change in the transmission spectra for every 1 °C was measured which defines the resolution of the proposed sensors. The sensitivities of these optical fiber sensors were calculated from the transmission spectra where average change in transmission spectra was recorded as 3.5% with every 1 °C for blue, 3% for red and 1% for orange-yellow thermochromic powders based optical fiber tip sensors. Among these sensors, highest and lowest sensitivities of 3.5% and 1% were recored for blue and orange-yellow tip sensors, respectively. All the transmission and reflection (heating–cooling) measurements are presented with the difference of 2 °C to avoid the overlapping of curves for clear interpretation of experimental data.

All reported sensors were measured multiple times to ensure the repeatability of the sensor and reproducibility of the experimental data. The transmission data for the blue optical fiber tip sensor was obtained for three continuous cycles and presented in Fig. S1A. It was found that the sensor is completely reusable with a rapid response time. The reusability of the blue sensor was also checked using a green laser. The transmitted power of laser was measured using photodetector for temperature change from 25 to 35 °C and can be seen in Fig. S1B. A fair agreement of sensor reusability was obtained. Moreover, the developed optical fiber sensors offer both transmission and reflection modes sensing mechanisms. However, variations in transmission spectra are more prominent than reflection-based sensing mechanisms. Nevertheless, as demonstrated, the optical fiber probe sensors are sensitive to low-temperature ranges. Therefore, they are potential candidates to monitor the temperature in biological environments such as beneath the skin, biological cells and tissues, and cell cultures^52^.

## Key challenges, solutions, and limitations

3D printing is a dynamic technical platform with excellent potential in numerous areas including biomedical applications. Herein, the demonstrated novel single droplet 3D printing method produced excellent results of sensor fabrication based on optical fibers and fiber tips. All the 3D printing technologies face challenges and optimization issues including our fabrication process. The main challenge in this work is the manual control of the printing process providing limited control over layer thickness, leading to irregular interfaces for longer optical fibers. However, a computer-controlled movement system could control the exact layer thickness during 3D printing. As laser source power is constant, the layer thickness during fabrication is varied and is gradually reduced for the sensor with longer length. Similarly, it could be addressed using a variable laser power source and maintaining the same exposure time. Moreover, for colored materials including thermochromic powders, an optimized concentration must be used to have minimum impact on collimated laser light transmission during the fabrication process, otherwise, obstructed curing light may result in bead formations rather than optical fibers. The main limitation of the current printing setup is its utilization for the fabrication of optical fiber and fiber tips only. However, by integrating complex and computer-controlled schemes with the current fabrication process, other complex shape structures with precise dimensions might also be possible.

## Conclusions and prospects

In this work, a novel single droplet 3D printing process was successfully used to fabricate transparent and thermochromic optical fiber sensors. Three thermochromic powders, i.e. blue, red, and orange to yellow were successfully incorporated into the polymer matrix. The fabricated thermochromic sensors exhibited an adequate optical response based on temperature ranges and variations. The fiber tip sensors (blue and red) showed a color to colorless transition as an optical response at the temperature of 25–35 °C, while the orange to yellow tip sensor showed color to color transition as optical response in the 25–31 °C temperature range. The orange to yellow tip sensor also provided a target-specific wavelength dependent optical response. The transmission and reflection intensities increased with the temperature increase for unicolor and dual color-based thermochromic optical fiber sensors and vice versa. The developed optical fiber sensors also responded adequately during reflection spectra measurements. The developed sensors are suitable for applications in both transmission and reflection sensing modes based on the experimental results.

The developed thermochromic sensors are promising candidates in thermal sensing applications such as aquatic environments. Additionally, these sensors might be used for strain sensing properties based on bending tests. A variety of photocurable materials (type and temperature range) could be used using this process. Moreover, optical tunable nanostructures such as Aztec structures, could be imprinted on optical fiber tips using the current fabrication process for biosensing applications. Overall, it can be said that 3D printing of stimulus-responsive optical fibers has enormous potential in sensing applications, although they still need more research and improvements. Additionally, multi-functional sensing based on optical fiber sensors is expected to have a significant role in targeted clinical applications.

## Methods

### Materials

A photocurable transparent resin material was developed comprising of three main components: Hydroxyethyl methacrylate (HEMA) as monomer, polyethylene glycol diacrylate (PEGDA) as crosslinker with molecular of 2000 Da, and trimethybenzoyl diphenylphosphine oxide (TPO) as photoinitiator (supplied by Sigma Aldrich). A commercially available silicon elastomer Sylgard 184 prepolymer and deionized water were used for swelling and cleaning (supplied by Sigma Aldrich). Blue and red unicolor thermochromic pigment powders were acquired from Amazon vendor in UAE under brand QIYMAR, with 4351144075 production reference. Similarly, dual color (orange to yellow) thermochromic pigment powder of LET’ RESIN brand was also acquired from UAE-based Amazon.

### Preparation of resin and temperature-sensitive resin

**Figure 7:**
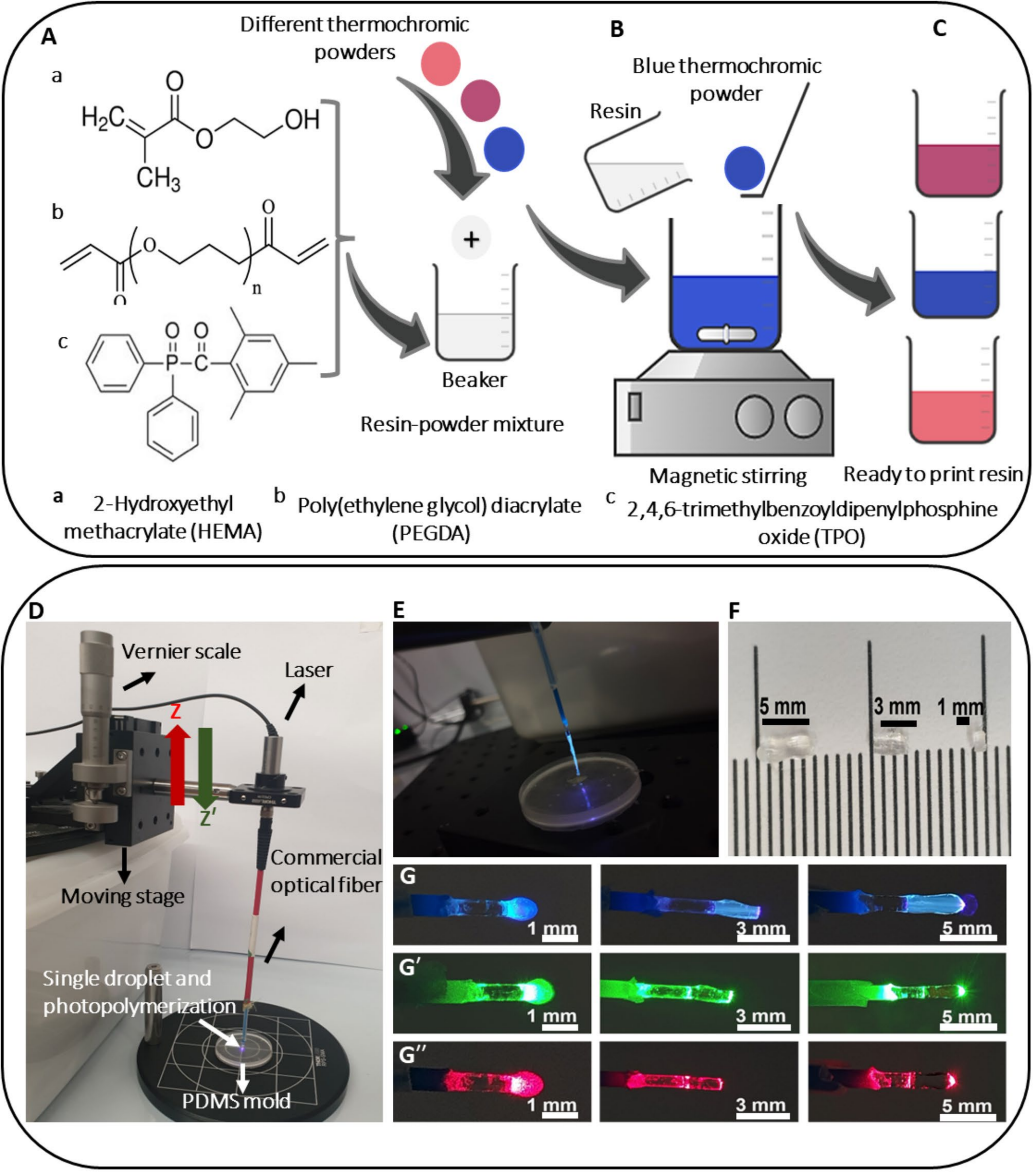
Preparation of printing material, fabrication process, and testing specimens; (**A**) chemical structures of the components of transparent HEMA-based resin (**B**), mixing thermochromic pigment micro-powders (blue, red, and orange to yellow), (**C**) storing ready to print resin material, (**D**) experimental setup of single droplet 3D printing process, (**E**) digital image of the transparent optical fiber during the printing process, (**F**) 3D printed transparent optical fibers of three different lengths (1, 3 and 5 mm), and (**G,G′,G′′**) represents digital images of 3D printed optical fibers transmitting three different lasers; blue (405 nm), green (532 nm), and red (650 nm).

The polymer-based optical fibers were fabricated via a novel 3D printing process by resining three main components of HEMA, PEGDA, and TPO at room temperature. Initially, HEMA and PEGDA were mixed in 1:1 volume ratio in a beaker and then 5 wt.% TPO was added as a photoinitiator to prepare HEMA-based photocurable resin. The chemical structures of these components are provided as shown in Fig. 7A. Initially, upon exposure to UV light, TPO generates free radicals where the energy level of the π* state is lowered by the phosphorus atom next to the carbonyl group, pushing the maximum of the n to π* transition toward 400 nm^53^. Then, these free radicals attack the c-bonds in PEGDA, and the reaction generates PEGDA crosslinking network^54^. Eventually, the PEGDA network reacts with HEMA to form PEGDA/HEMA hydrogels^55^. The amount of TPO utilized ensured an adequate 3D printing process and suitable optical properties simultaneously. HEMA was used as a monomer in resin solution and is reported to be a flexible and biocompatible polymer suitable for in vivo and in vitro testing in clinical applications^6,56^. In addition to assisting in crosslinking HEMA, PEGDA is a long-chain and flexible polymer that can create photopolymerizable polymers. TPO was responsible for initiating photopolymerization reaction with 385–420 nm wavelength range during UV light exposure.

Finally, 1 wt.% thermochromic pigment powder of each color was added to HEMA-based resin and magnetically stirred for 30 min to ensure homogenized resin solution (Fig. 7B,C). The optimum quantity of each powder was selected to produce an adequate optical and thermal response from fiber tips without affecting the printing process. Three powders named blue, red, and orange to yellow were used, and samples were named based on imparted colors to the resin solution. For instance, transparent sample is (HEMA + PEGDA only), blue sample is (HMEA + PEGDA + blue powder), red sample is (HEMA + PEGDA + red powder), and the orange to yellow is resin (HEMA + PEGDA + orange to yellow powder). TPO assisted all the transparent and pigmented colored samples as the photoinitiator.

### Single droplet 3D printing process

The experimental setup of the fabrication process consisted of a large-area rotating breadboard (RBB300A/M, 360° continuous, Ø300mm, hand-operated rotation, Thorlabs), linear translational stage (Axes of travel Z-Z′, maximum stage travel 25 mm, 20 µm stepsize, Newport), laser mount cage plat (CP02/M), connecting rod, collimated blue laser module (CPS405, 405 nm wavelength and 4.5 mW power, Thorlabs), and commercial optical fiber (multimode optical fiber (M35101, Ø1000 μm, 0.39 NA, Thorlabs) illustrated in (Fig. 7D). First, the translational stage was mounted on rotating breadboard that allows mounting stage with 360° freedom in our experimental setup. The translational stage has a maximum travel distance of 45 mm allowing us to fabricate optical fibers of the same length. The translational stage movement of 1 mm on a linear scale is divided into 50 divisions on the rotational Vernier scale. Thus, the rotational scale allows moving optical fiber with a stepsize of 20 µm in the Z or Z′ direction. The cage plate was used for mounting the blue laser (on the top) and an optical fiber coupler (at the bottom). The then cage plate was mounted onto a translational stage using a connecting rod. The blue laser has wavelength of 405 nm with 4.5 mW power and it was in the curing wavelength range of photoinitiator used (385–420 nm).

A sylgard 184 prepolymer having 1:10 ratio by weight of curing agent to base is poured onto the master pattern with circular cavity and then cured at room temperature for 24 h to crosslink the polymer. The cured PDMS mold is peeled from the master pattern and used as resin droplet tank. PDMS mold with circular cavity was filled with 15 µm of photocurable resin droplet by using a pipette and placed below the commercial optical fiber, before initiating the fabrication process. The optical fiber was brought closer to the resin droplet using a Vernier scale micrometer, ensuring that the fiber tip merely touched the upper surface of the droplet. Silanization process was used to increase the adhesion between the hydrogel and silica optical fiber. Silanization provides the chemical bonding between the silica and hydrogel matrix. 3–(trimethoxysilyl) propyl methacrylate was used as the coupling agent for the silanization procedure. A silanization solution was prepared by mixing 1 ml of 3–(trimethoxysilyl) propyl methacrylate and 50 ml of acetone together. The optical fiber tip was immersed in the prepared solution for 30 min and left for 24 h drying^57,58^. The 3D printing process was initiated by switching on the laser, allowing light to pass through the commercial optical fiber, towards it resin dipped tip. When laser light reached the resin material, photopolymerization reaction was initiated, and resin started curing onto the optical fiber tip. Based on the desired length, optical fiber was lifted periodically with specific time intervals in the Z-direction. Thus, transparent optical fibers with suitable lengths were produced with proper stepsize (layer thickness) and curing time intervals (Fig. 7E–G′′). In the photopolymerization process, initial layer thickness was high for sensors with more than 1 mm length and then reduced for the constant curing time intervals. Therefore, more interfaces were observed for longer length sensors due to varying layer thicknesses. Moreover, high concentrations of thermochromic powders reduced the curing light transmission during the fabrication, which can lead to bead shaped fiber tip formation. However, comparatively longer functionalized tips can be produced using lower concentrations of functional materials or variable laser power source. All the transparent and thermochromic optical fiber tips were fabricated using the same procedure. To fabricate optical fiber and fiber tips with specific lengths, the relevant printing parameters were studied including, Vernier scale stepsize movements (layer thicknesses) and curing time intervals, as summarized in Table 1**.**

**Table 1:** Optical fiber types and process parameters of single droplet 3D printing process.

Optical fiber types	Optical fiber length (mm)	Layer thickness (µm)	Curing time/layer in seconds (s)	Number of layers
Transparent optical fibers	1	500	30	2
	3	400	20	6
		200	20	3
	5	400	20	6
		200	20	13
Blue optical fiber tip	1	500	30	2
Red optical fiber tip	1	500	30	2
Orange to yellow optical fiber tip	1	500	30	2

### Materials characterization

The printed optical fibers and fiber tips were investigated using their physical, microstructural, and optical characterizations. The microstructural investigation of three thermochromic powders and cross-sectional imaging of the printed optical fiber tips was carried out using field emission gun scanning electron microscopy (FEG-SEM; JSM-7610F). The SEM operating parameters included the working distance of 8 mm, accelerating voltage of 4 kV, and probe current of 7 nA. Before the.

SEM characterization, powders and printed samples were gold coated with the direct current sputtering machine (JEOL JCM-3000FC). The coating parameters of 30 mA current, 40 s coating time, and 4.5 Pa vacuum created gold coating of 3 nm to avoid charging effect during characterization. The embedded thermochromic powders were studied from the cross-sections of mechanically fractured surfaces of printed fibers. The surface morphology and optical transmission properties of the printed optical fibers and fiber tips were investigated using an optical microscope (ZEISS Axio Scope 1, Germany). The optical transmission and reflections spectra of materials including HEMA-based transparent resin, thermochromic resins, 3D printed transparent, and pigmented optical fibers were recorded using a UV–vis spectrometer (USB 4000 + , Ocean Optics) having 400–1100 nm wavelengths measuring range. The spectrometer detects targets at spectral regions restricted to visible and near-infrared (VNIR) regimes^59^. The crystallinity of utilized thermochromic powders and any second phase formation during the printing process were confirmed by X-ray diffraction (XRD) analysis using D2 PHASE diffractometer (Bruker, Germany)^6,26,44^. The XRD experiments were conducted in 5–60° two theta range, with 2.49°/min scan speed and 0.02° incremental step size. The diffractometer has a Lynxeye detector that operates in 1D mode and employs CuKα radiations of wavelength 1.54 Å at 30 kV.

### Measurement setups

The following four experimental setups were used to thoroughly investigate optical and thermal responses of the 3D printed fibers and functionalized tips.

#### Transmission measurements

The transmission measurement setup was made of an optical bench, white light source, 2-terminal multimode optical (M35l01, Ø1000 μm, 0.39 NA, Thorlabs), connectors, UV–vis spectrometer (USB 4000 + , Ocean Optics), and cage plate with an embedded lens. The components mentioned were properly connected as shown in Fig. 2A. The white light transmitted through optical fiber sensor was collected by a focusing lens and then refocused into a spectrometer. Before recording their respective transmission spectra, commercial optical fiber was used as a reference for the printed sensors (transparent and pigmented). Dry samples of transparent optical fibers and pigmented tips were tested for their transmission spectra, and separate transmission spectrum was recorded with respect to wavelength for each material. HEMA is a hydrogel and allows itself to swell by absorbing oxygen through water molecules^27,60^. Therefore, the swelling behavior of the printed sensors (transparent and pigmented) was evaluated by submerging them in Petri dish containing 3 ml of DI water. The printed sensors swelled over time, and each sensor’s transmission spectrum was recorded over specific time intervals against the transmitted wavelength. Thus, a relationship between sensor swelling and variations in the spectrum of each sensor is established. 3 ml DI water was used in the experiment, and it was found that altering the amount of liquid (changing the height) had no impact on the sensors’ functionality^42^. The tips of commercial optical fiber and fabricated sensors were kept constant at 2 mm distance from the cage plate equipped with a lens to collect the transmitted light for transmission measurements. To safely detach printed fibers and fiber tips from the commercial optical fiber at the end of the experiment, the sensors were submerged in DI water solution for at least 1 h.

#### Reflection measurements 

The fabricated fibers were also tested in reflection mode of operation. The reflection measurement setup consisted of an optical bench, white light source, 3-terminal multimode optical (M35l01, Ø1000 μm, 0.39 NA, Thorlabs), connectors, UV–vis spectrometer (USB 4000 + , Ocean Optics), and a highly reflecting surface (mirror, Thorlabs). The components were connected correctly, and a mirror was placed 2 mm below the transparent optical sensors to facilitate reflection measurements. The schematic diagram of the experimental setup is provided in Fig. 2B, and the reflection spectrum of each transparent optical sensor was recorded with respect to wavelength.

#### Optical power measurements 

The transmitted power of several lasers, through the fabricated transparent optical fibers, was measured using another customized optical setup. The schematic illustration of the experimental setup is provided in Fig. S2 (Supporting Information). The experimental setup consisted of an optical bench, three lasers including blue (405 nm, 4.5 mW), green (532 nm, 4.5 mW), and red (650 nm, 4.5 mW), optical power meter (PMD100D, 100 pW-200W range), multimode optical fiber (M35101), and photodetector (S120C, 400–1100 nm, 50mW). All the components utilized in this experiment were purchased from Thorlabs.

#### Transmission and temperature measurements

The thermal sensing functionality of the optical fiber probes was assessed using the same experimental setup used for transmission and swelling measurements, except for connecting the thermometer to Petri dish, as shown in Fig. S3 (Supporting Information). Before starting temperature measurements, sensors were immersed in DI water for at least 3 h to ensure complete swelling of the developed sensors. The temperature measurements were carried out in hot DI water to confirm homogenized heat distribution in the sensor^6,26^, and an attached thermometer monitored the water temperature. The starting temperature of pigmented sensors was 25 °C and was gradually increased by 1 °C to 35 °C and 31 °C for unicolor and dual color powders, respectively. The tip sensors were colorful (blue and red) at 25 °C and turned transparent for unicolor powder at 35 °C. However, the dual color tip sensor was orange at 25 °C and turned yellow at 31 °C^6,26,61^. The transmission spectra of pigmented fiber tip sensors were recorded with respect to wavelength for each degree variation of DI water temperature. Thus, a correlation between the optical transmission spectrum and temperature was established for each sensor. The optical fiber sensors were washed and separated, as mentioned earlier.

#### Reflection and temperature measurements 

Similar experimental setup components based on reflection and temperature measurements were used except 2-terminal multimode optical fiber was replaced with 3-terminal multimode bundle fiber, as shown in Fig. S4 (Supporting Information). The printed thermochromic sensors were immersed in petri dish filled with 3 ml of hot water, and the reflection spectrum of each sensor was recorded with respect to the wavelength at specific temperatures. The water temperature was monitored by an attached thermometer to the experimental setup. However, before reflection experiments, developed sensors were immersed in DI water for at least 3 h to ensure complete swelling of sensors. Thus, similar correlation was established between variations in reflection spectra and the temperature rise/fall of the pigmented fiber sensors. The same washing and separating protocols were used before starting the following experiment.

## Supplementary Information

Supplementary Figures.

## Data Availability

The optical spectroscopic data analyzed during the current study is available from the corresponding authors on reasonable request.
