# Supplementary material for: UV polymerization fabrication method for polymer composite based optical fiber sensors
Source: Sci Rep. 2023 Jul 4;13:10823. doi: 10.1038/s41598-023-33991-6 (PMC10319730; doi:10.1038/s41598-023-33991-6)
Supplement: Supplementary file 1 — Supplementary Figures. [file 41598_2023_33991_MOESM1_ESM.pdf]

UV Polymerization Fabrication Method for Polymer Composite based Optical Fiber Sensors

Israr Ahmed, Murad Ali, Haider Butt

Department of Mechanical Engineering, Khalifa University of Science and Technology, Abu Dhabi, 127788 UAE

Corresponding Authors: Haider Butt (haider.butt@ku.ac.ae); Murad Ali (murad.ali@ku.ac.ae)

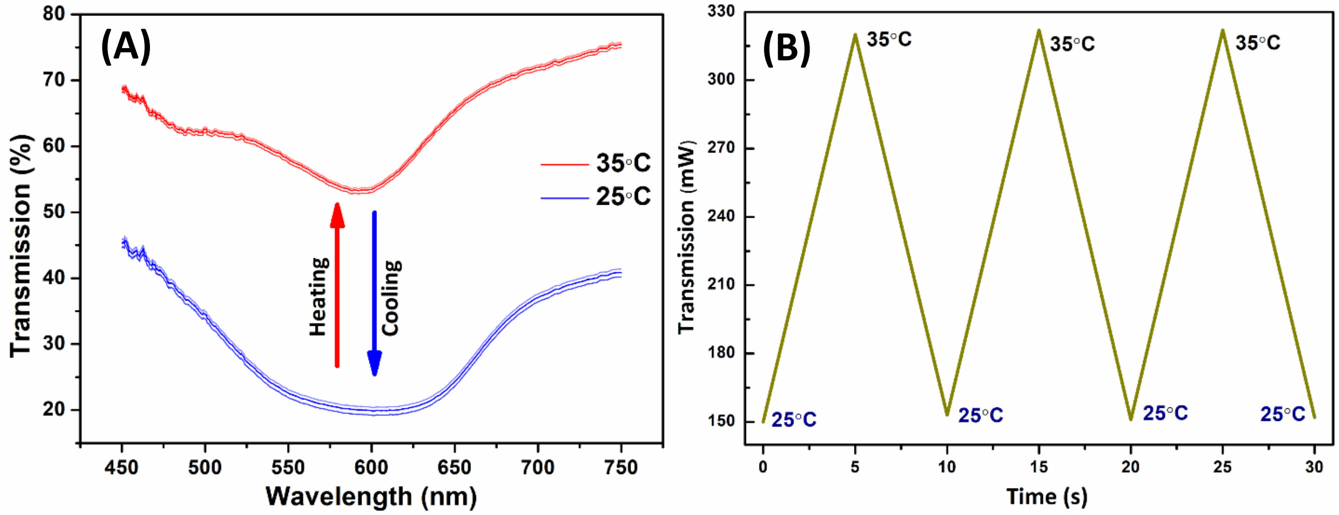

Figure S1: (A) Transmission spectra of blue optical fiber tip sensor for three continuous measurement cycles for temperature change from 25°C to 35°C. (B) Three measurement cycles for blue optical fiber tip sensor using green laser for temperature change from 25°C to 35°C

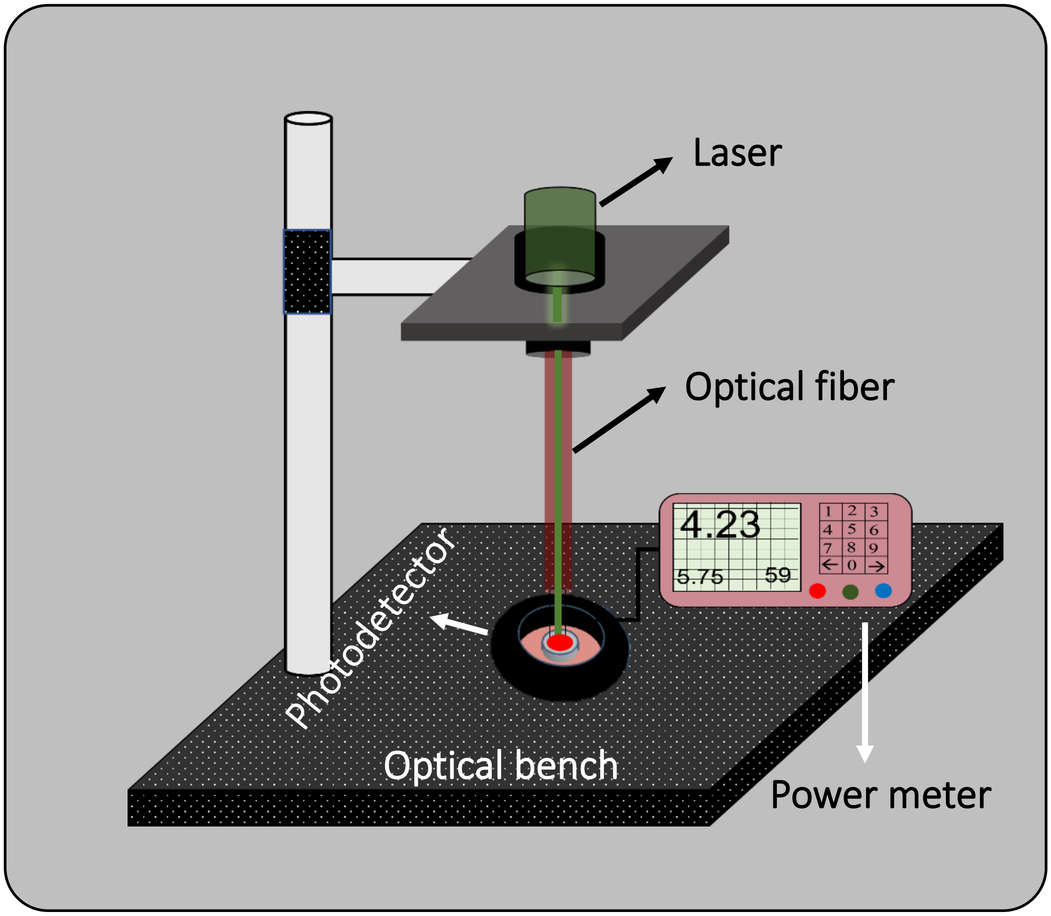

Figure S2: Experimental setup of transmitted powder measurement for transparent optical fiber sensors.

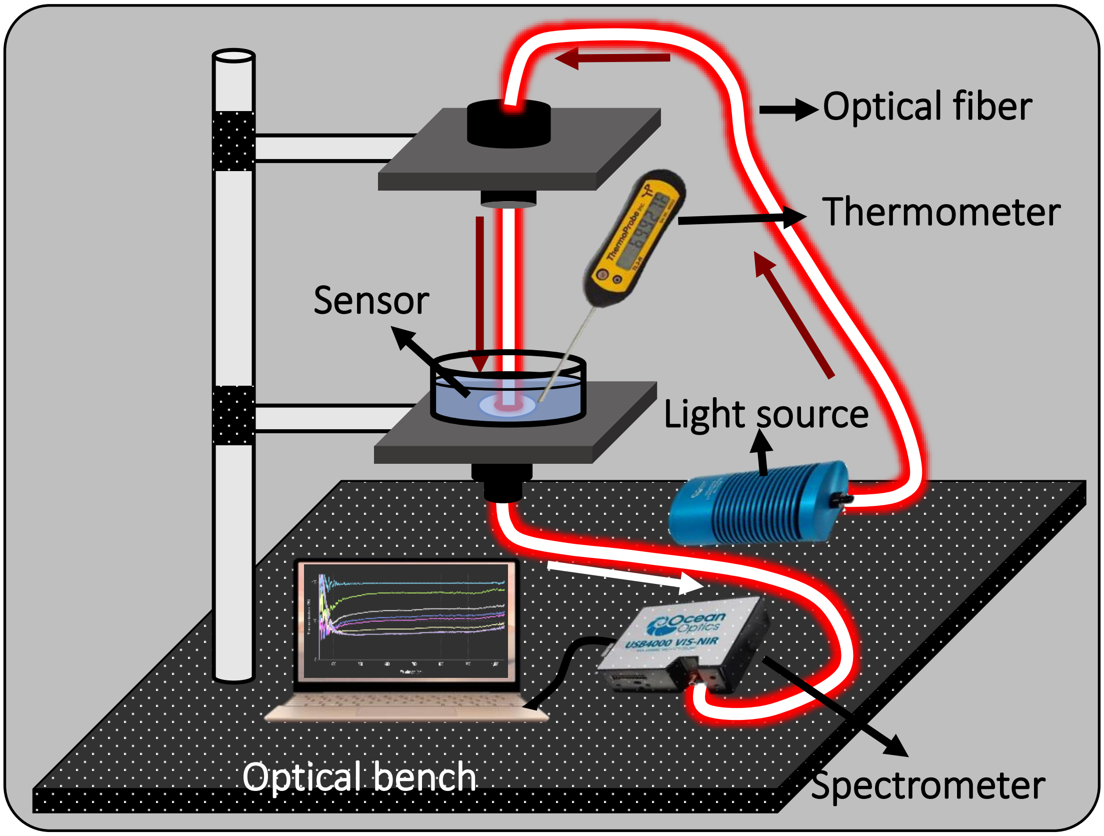

Figure S3: Schematic of transmission measurement setup utilized to measure the thermal response of optical fiber sensors.

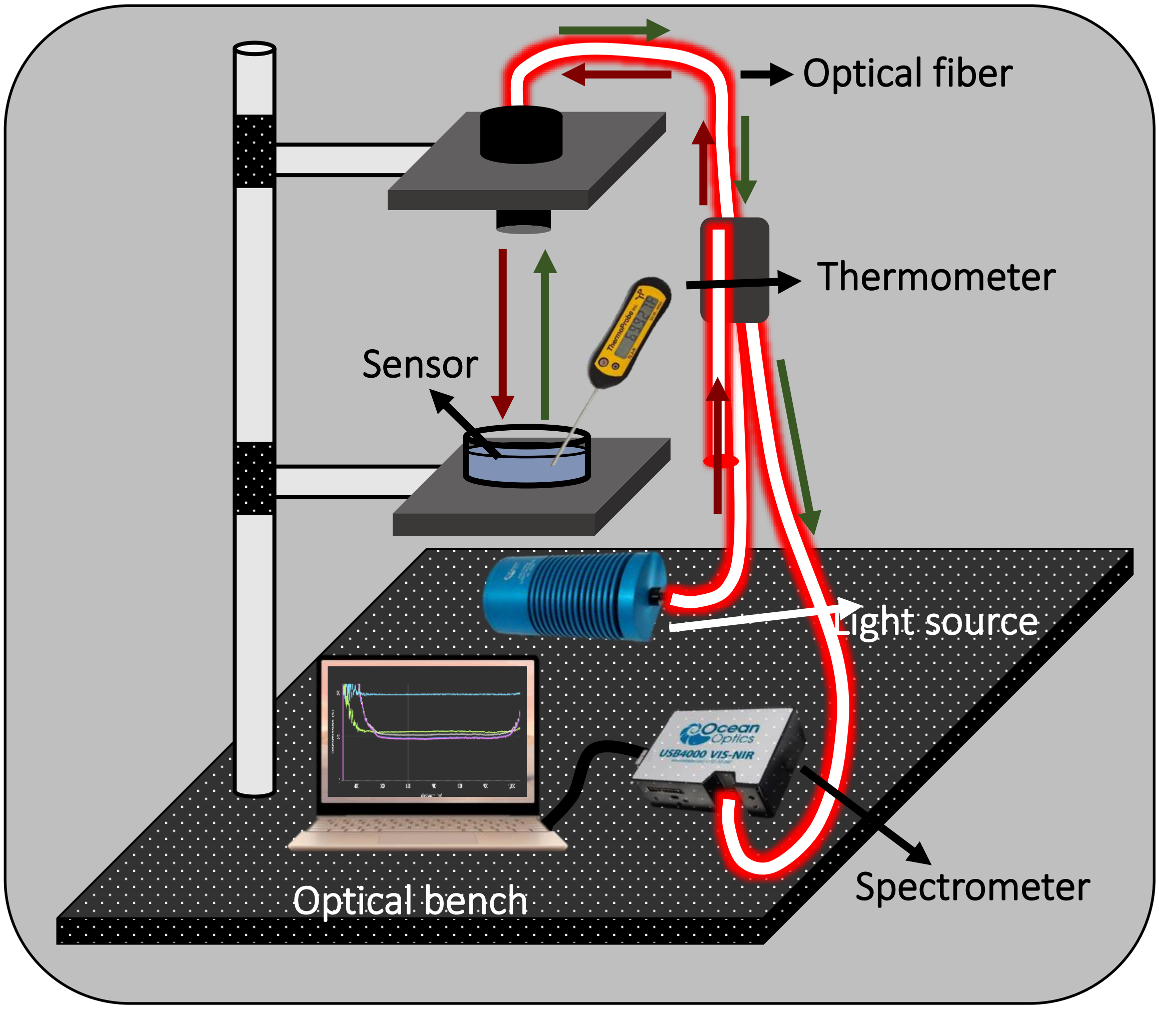

Figure S4: Schematic of reflection measurement setup utilized to measure the thermal response of optical fiber sensors.
